# Differences in gaze anticipation for locomotion with and without vision

**DOI:** 10.3389/fnhum.2015.00312

**Published:** 2015-06-08

**Authors:** Colas N. Authié, Pauline M. Hilt, Steve N'Guyen, Alain Berthoz, Daniel Bennequin

**Affiliations:** ^1^Laboratoire de Physiologie de la Perception et de l'Action, UMR 7152, Collège de France, Centre National de la Recherche ScientifiqueParis, France; ^2^UFR de Mathématiques, Équipe Géométrie et Dynamique, Institut de Mathématiques de Jussieu, Université Paris Diderot-Paris 7, UMR 7586Paris, France

**Keywords:** eye movement, nystagmus, OKN, human locomotion, anticipation

## Abstract

Previous experimental studies have shown a spontaneous anticipation of locomotor trajectory by the head and gaze direction during human locomotion. This anticipatory behavior could serve several functions: an optimal selection of visual information, for instance through landmarks and optic flow, as well as trajectory planning and motor control. This would imply that anticipation remains in darkness but with different characteristics. We asked 10 participants to walk along two predefined complex trajectories (limaçon and figure eight) without any cue on the trajectory to follow. Two visual conditions were used: (*i*) in light and (*ii*) in complete darkness with eyes open. The whole body kinematics were recorded by motion capture, along with the participant's right eye movements. We showed that in darkness and in light, horizontal gaze anticipates the orientation of the head which itself anticipates the trajectory direction. However, the horizontal angular anticipation decreases by a half in darkness for both gaze and head. In both visual conditions we observed an eye nystagmus with similar properties (frequency and amplitude). The main difference comes from the fact that in light, there is a shift of the orientations of the eye nystagmus and the head in the direction of the trajectory. These results suggest that a fundamental function of gaze is to represent self motion, stabilize the perception of space during locomotion, and to simulate the future trajectory, regardless of the vision condition.

## 1. Introduction

Human locomotion is a complex task, which requires a multisensory integration, and a variety of motor and cognitive controls. Many studies (Grasso et al., 1996, 1998; Patla and Vickers, 1997; Hollands et al., 2002; Hicheur and Berthoz, 2005; Hicheur et al., 2005; Bernardin et al., 2012) have highlighted the important role of head direction and gaze (i.e., the direction of optic axis in space) in guiding, controlling, and anticipating locomotor trajectory. Visual perception during locomotion helps avoiding perturbations and preparing motor adjustments (see Higuchi, 2013 for a review). Pozzo et al. (1990) have shown that the head is stabilized in rotation in the vertical sagittal plane and suggested that this stabilization helps visual perception and allows a matching of visual and vestibular information, relevant to motor control, space perception and updating. They interpreted that the stabilization of the head creates an inertial guidance platform serving as a mobile reference frame for the coordination of the multiple body segments during locomotion. In the same study, they also showed that the plane of stabilization was controlled by the gaze and then proposed that the gaze was used as a part of the reference frame. Gaze is also known to anticipate during arm reaching (Paillard, 1982; Jeannerod, 1988; Flanders et al., 1999), grasping and objects manipulations (Johansson et al., 2001). Anticipatory behavior by gaze and head has also been observed in other human movements (Land and Lee, 1994; Land et al., 1999; Hollands et al., 2002; Wilkie and Wann, 2003; Marigold and Patla, 2007).

During locomotion, gaze exhibits a manifold of functional behavior, as for instance, fixations of several types. Patla and Vickers (2003) proposed to distinguish “travel fixations,” where the gaze tends to stay parallel to a fixed direction in space, and “point fixations,” in which gaze rotates in space to remain fixed on focalized locations in the environment. There is also a diversity of saccadic eye movements: normal, express, micros, catch-up saccades and series of hypometric saccades. In addition, the human visual system has the ability to use visual pursuit in order to follow a slowly moving target in the environment. Moreover, during locomotion, a functional nystagmus occurs (Solomon and Cohen, 1992a,b for monkeys; Grasso et al., 1998; Imai et al., 2001 for humans). This nystagmus comprises a succession of alternating quick and slow phases of the eye. The slow phases stabilize gaze in space or stabilize the visual scene.

The nystagmus was extensively studied in laboratory, without resorting to participants' self-motion (e.g., Niemann et al., 1999; Kaminiarz et al., 2009). When visual stimuli are flowing or when the subject is rotating, a nystagmus occurs. Its quick phases are known to allow for a redirection of the gaze toward the appearing visual scene. This phenomenon has not yet been studied in locomotion.

Recently, the anticipation of locomotor trajectory by gaze and head direction has been precisely quantified in tasks of path reproduction, with complex trajectories including changes of direction and curvature (Kadone et al., 2010; Bernardin et al., 2012). The authors reported a systematic behavior: the direction of gaze anticipates the direction of the head, which itself anticipates the trunk, the feet and the direction of the trajectory. However, head rotations have proven neither necessary nor sufficient to steering control (Cinelli and Warren, 2012). But in natural conditions, along curved trajectories, spontaneous anticipatory rotations of the head are nevertheless observed.

A large part of visuo-motor coordination, involved in the perception and control of self motion, can be explained on the basis of information extracted from the optic flow (*cf*. Authié and Mestre, 2011). In particular, gaze and head anticipations observed in humans while moving could take part in the extraction of an optimal set of information in the optic flow field (Authié and Mestre, 2012). Moreover, head anticipation could assist the global gaze orientation toward visual goals (Reed-Jones et al., 2009; Mestre and Authié, 2012); see also (Shepherd, 2010) for a discussion of gaze-following behaviors in humans and other animals.

However, Grasso et al. (1998) reported the persistence of anticipatory movements of the head and gaze during locomotion around a corner without vision. Later, Courtine and Schieppati (2003b) reported head anticipation during locomotion without vision on longer trajectories with constant curvature. These results proved that gaze and head anticipation do not depend only on visual cues. Grasso et al. (1998) proposed that this behavior corresponds to a feed-forward navigation control system to anticipate motor events. In addition, Berthoz (1997) suggested the possibility that gaze movement is used in a mental simulation of the locomotor path. This hypothesis is supported by (*i*) the ability to memorize and trace, while being blindfolded, circular trajectories (Takei et al., 1997) or triangular paths (Glasauer et al., 2002), and (*ii*) the fact that human subjects walking backwards on circular paths orient head and gaze in such a way that the opposite vectors anticipate the body motion (Grasso et al., 1996, 1998; Courtine and Schieppati, 2003a for the head).

Importantly, Solomon and Cohen (1992a) established that monkeys walking on circular trajectories in the dark exhibit head and eye nystagmi, with quick phases in the direction of running. The authors compared this nystagmus with the corresponding behavior in the light. They showed that this nystagmus is not produced by passive rotation. They came to the conclusion that there was an innate pattern of eye-head coordination supporting gaze compensation during locomotion, and attributed this pattern to a velocity storage mechanism in the vestibular system. However, while their data show that in this coordination pattern, gaze is always in advance with respect to the head direction, that is itself in advance with respect to the heading displacement, this critical point is not at all discussed in the article.

This anticipatory behavior in monkey raise multiple questions. Is anticipation of human subjects maintained without vision? What is the function of this anticipation? Is it related to trajectory planning? To answer these questions, we need to compare gaze and head behaviors of humans during free locomotion on complex paths with curvature changes, in the dark and in the light. In the present experiment, participants had to walk along memorized trajectories.

Our first prediction was that gaze and head anticipation of the locomotor trajectory persist in the dark, even in this more complex context. This prediction was based on the assumption that, in addition to visual functions, the gaze anticipation has at least three other functions:

- a motor anticipation (Grasso et al., 1998),
- a mental simulation of the trajectory (Berthoz, 1997; Vieilledent et al., 2003),
- an active process based on vestibular and proprioceptive information, contributing to spatial perception during self motion (*cf*. Reuschel et al., 2012).

And these three functions should remain active in total darkness.

Our second prediction was that the gaze anticipation may have different properties in the light and in the dark. This prediction was based on several reasons: (a) the mechanisms in the dark and in the light rely on different sensory inputs; (b) some functions of gaze and head are specific to vision, such as the maximization of visual information, visual attention, and optic flow stabilization, while some are specific to an absence of vision, such as the maximization of vestibular and proprioceptive information and velocity storage; some others are common to both conditions, such as motor planning, trajectory simulation and spatial perception. One might as well-conjecture that all these functions are reflected by different kinematic characteristics of gaze and head movements. For instance, fixation and pursuit would be reflected in a smooth component of gaze kinematic and saccadic explorations by a discrete component.

Therefore, we conjectured that the main difference between dark and light observations shall be a different type of gaze anticipation to be determined more specifically, but with a preservation of the nystagmus, with similar amplitude and frequency.

## 2. Materials and methods

### 2.1. Participants

The participants were all members of the LPPA Laboratory, and not informed of the purpose of the experiment. Overall 12 persons participated in the experiment. Two amongst them were withdrawn from the pool of participants, for not respecting experimental instructions. We were then left with 4 female and 6 male participants [μ(age) = 30, σ = 5.2]. All participants had a normal vision or corrected to normal by contact lenses. No participant reported any present or past trouble regarding their motor or perceptive skills. The participants signed an informed consent form (AFSSAPS: 2009-A00739-48/CPP: 58-09) approved by the ethical committee in accordance with the standards established by the Declaration of Helsinki.

### 2.2. Recording apparatus

Motion capture of the whole body kinematics has been realized through a VICON system (VICON Motion Systems Inc., Los Angeles, 13 cameras, 120 Hz). Participants wore a tight black suit allowing to stick the VICON markers as close to the participant's body as needed. The 43 markers were placed according to the “Plug-in Gait” model of the VICON system (VICON, V-1.7). To approximate the position of the head, four additional markers were located over the right and left temples on the back and at the front of the head. The positions of the markers were recorded, reconstructed, and labeled using VICON iQ software.

**Figure 1:**
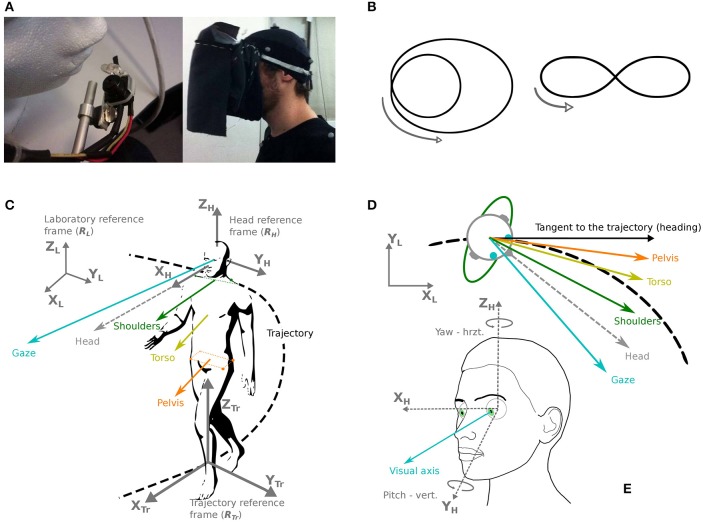
**Apparatus, trajectories, and definition of coordinates and studied body segments**. **(A)** Left: the eye-tracker camera used in the experiment. Right: the fabric screen used to prevent any source of light during the experiment. **(B)** Two trajectories were shown and required to be followed by the participants: a limaçon (left) and an elongated eight-like shape (right). **(C)** Chosen reference frames (gray): laboratory (*R*_*L*_), head (*R*_*H*_) and trajectory (*R*_*Tr*_). The considered segments are: gaze (blue), head (gray dotted line), shoulders (green), torso (yellow), pelvis (orange). **(D)** Direction of the various segments in the horizontal plane, with respect to the trajectory and its tangent. **(E)** Definition of the studied eye movements (visual axis, blue), in azimuth and yaw.

The recording of eye movements has been performed thanks to a head-mounted video eye-tracker (Mocaplab, Paris, 50 Hz). The camera was located under the participant's right eye, but not obscuring a too important part of the participant's field of vision (Figure 1A). The camera output was transmitted to a VAIO P computer (mass: 600 g), tied on the back of the participant.

### 2.3. Calibration

Before each trial, several calibrations must be performed to insure a good definition of the room space and of the participants' movements for both the eye-tracker and motion capture system. To determine the center of rotation of each eye with respect to the head markers, we asked the participants to visually align two VICON markers fixed on a stick with each eye (separately) and to keep rotating their head at the same time. In order to calibrate the whole system (eye-tracker and motion capture), we used a 145 cm wide and 100 cm high elliptic calibration grid, composed of 25 visual targets. It was placed in front of the participant while sitting on a chair at a distance that allowed full view of the grid without moving the head. The participant had to look at each target, in order to match the pupil position recorded by the eye-tracker and the position of the displayed targets from the orbit position. To check the eye-tracker headband stability on the head during the trials, this calibration was performed at the beginning and at the end of each experiment.

Additionally, the participants fixated one extra target on the floor at the beginning and at the end of each trial, to check eye-tracker positioning and calibration quality.

Temporal synchronization of eye-tracker and VICON signals were performed offline. Before each recording, a clap board was activated. A set of VICON markers were fixed to it, and its closing triggered a LED at the top of the eye-tracker camera. The triggering signal was therefore simultaneously received by both systems.

### 2.4. Task

The main task was to walk at normal pace along two predefined trajectories: an eight-like elongated shape and a limaçon (Figure 1B). These trajectories were chosen so that behavioral changes could be studied, in relation with trajectory characteristics: direction change (eight shape) and variations of curvature (limaçon). The two trajectories were shown to the participants on a sheet of paper; then an example was given in the testing room by the experimenter before the beginning of the first trial. The participants received the instructions to reproduce the trajectory announced at the beginning of the trial, as naturally as possible and at a preferred speed, the important parameter being the shape of the trajectory to follow, without necessarily coming back to the initial position.

The participants were expected to reproduce the two trajectories under two different visual conditions: in light and in darkness. In order to avoid any source of light in the darkness condition, a fabric screen, opaque to light, had been fixed to the eye-tracker, allowing to put the participant into full obscurity easily and without moving the eye-tracker (Figure 1A). This screen weighted 84 g and was not in contact with the participant's face or the eye-tracker.

Once equipped, the participant practiced walking in the dark during a dedicated accommodation phase. The eye-tracker was not yet tied to the participant's head, so as to minimize the inconvenience for the participant. During the training, the participants were asked to follow a rectangular trajectory of about the dimension of the recording area, first in light, and at the speed of their convenience. This was done again afterwards without vision (fabric screen on, lights off). The accommodation phase lasted between 2 and 5 min, and stopped when the participant was able to walk at normal gait in the dark, while staying in the VICON recording area. The trajectory followed during the training phase was different from the one followed during the experiment.

The experimental sessions were divided into eight trials in darkness (four along each type of trajectory), and eight trials in light. To prevent the eye-tracker to slide around on the head by manipulating the fabric screen, we grouped the in-light trials together and likewise for the darkness trials. The experimental conditions were shuffled via a randomized order of the trajectories within a group of conditions (light/darkness), as well as randomized order of the condition groups (five participants started in darkness and five in light). Trials during which a participant went out of the capture area were started over. This happened mainly during darkness trials and on average three times per participant.

### 2.5. Experimenters and environment

To avoid the participants' attention to be diverted, most massive objects were put along walls and covered with black sheets. The two experimenters were sitting in the left corner of the testing room during free-sight trials (always at the same spot for all trials and all participants); during darkness trials, one experimenter was standing on the right and the other on the left, in case the participant came too close to a wall.

### 2.6. Data analysis

Data analysis has been performed with Matlab^©^ (Mathworks, Natick, US-MA). Statistical analyzes have been carried out with Matlab^©^ and Statistica^©^ (StatSoft, Tulsa, US-OK).

We defined the beginning and the end of the trajectories with a speed threshold and a number-of-steps criterion: if the pelvis speed was less than 0.1 m/s, the participant was considered stopped; if the detected trial comprised less than five steps, it was rejected. The detected start and stop times have been further checked manually.

### 2.7. Reference coordinate systems

In order to analyze participants' movements in space, as well as the relative movements of the most relevant body parts, six coordinate bases have been defined (Figures 1C,E):

- The reference frame of the laboratory. The unitary vector *X*_*L*_ is parallel to the width of the experimental room, the unitary vector *Y*_*L*_ is parallel to the depth of the room, and the unitary vector *Z*_*L*_ is oriented upwards, perpendicularly to the floor.
- A mobile reference frame attached to the head and with an origin at the isobarycenter of the markers situated on the participant's head. The direction of the unitary vector *X*_*H*_ is determined by the calibration (target fixation) as the line passing by the central target on the grid and the center of the head. The unitary vector *Z*_*H*_ is collinear to the gravity direction during the calibration phase and the unitary vector *Y*_*H*_, pointing to the left of the participant's head is defined as the vectorial product of *X*_*H*_ and *Z*_*H*_.

### 2.8. Most relevant parameters

The main directions of interest in this study are gaze, head direction and trajectory tangent (shoulder and torso directions were also considered, see Supplementary Results). For instance, the pelvis segment is represented by the vector originating from the isobarycenter of the four pelvis markers, and collinear to the line passing through the two right pelvis markers.

The head segment is represented by the *O*_*X*_ axis of the head reference system (Figure 1C).

With a sampling frequency of 120 Hz, computing speeds is relatively straightforward and a low-pass butterworth filtering with a cut-off frequency of 10 Hz allows to get rid of most of the noise due to the recording system (Bernardin et al., 2012).

In this article, the participant's trajectory will be reduced to the displacement of the isobarycenter of the pelvis (Bernardin et al., 2012). To compute the curvature of the participant's trajectory, we lowered the frequency cut-off down to 1 Hz in order to cancel the contribution of pace oscillations (Hicheur and Berthoz, 2005).

The instantaneous speed *v* of the participants has been defined by the pelvis isobarycenter speed. The trajectory curvature *c* has been defined as the instantaneous curvature of the pelvis trajectory, projected onto the ground plane. If (*x*, *y*) are the pelvis trajectory coordinates in the horizontal plane at instant *t*, the curvature is given by the following expression:

(1) $$c=\frac{x^{\prime }y^{\prime \prime }-y^{\prime }x^{\prime \prime }}{{\left({x^{\prime }}^{2}+{y^{\prime }}^{2}\right)}^{3/2}}$$

However, the reference direction in the horizontal plane for measuring angular anticipation, in time or in space, was chosen to be the direction of the pelvis, and not the tangent to the trajectory of the pelvis. We justified this choice by the higher stability of this direction when compared to the tangent, i.e., the angle of the tangent oscillates around the pelvis direction which has less oscillations in its variations. The results supporting this choice are detailed in the Appendix Supplementary Methods (Supplementary Figure 1).

### 2.9. Time shift and cross correlations

In order to examine the relative time shift of each body segment, we computed cross correlations of their horizontal orientation in space.

### 2.10. Decomposing the horizontal angle of gaze

We decided to analyze the position of gaze in the laboratory and above all to distinguish horizontal variations from vertical ones (Figure 1D). This distinction is based *a priori* on the assumption of a specific function of horizontal control (perpendicular to gravity).

### 2.11. Quick and slow phases detection

Before each gaze analysis, the phases corresponding to blinks were automatically excluded. Quick phases have been detected thanks to a method inspired by Van der Steen and Bruno (1995). First, experimental data points were identified as candidates if the following three criteria were fulfilled: a threshold speed of 30°/s; a minimal amplitude of one degree and an acceleration peak higher than 700°/s^2^. Beginnings and ends of the quick phases were then identified thanks to an algorithm maximizing the bi-dimensional distance between beginning and end of the quick phase. Each quick phase onset and offset were then manually checked to eventually correct the automatic algorithm. This process led to a loss of 8% of quick phases automatically detected. Only quick phases with the same direction as the heading, and separated by “slow phases,” were considered. Slow phases lasting more than a second which are extremely rare, were not analyzed (*cf*. Authié and Mestre, 2011).

### 2.12. Quick phase detection

At 50 Hz, the signal is not accurate enough to distinguish, based on the quick phase kinematics, a voluntary saccade from a nystagmus quick phase. The only detectable saccades are reverse-saccades, corresponding to quick eye movements in the opposite direction to the direction of displacement. The detection of reverse-saccades has been performed by considering the direction of variation of the quick jump slope as a function of the sign of the trajectory curvature. This detection was based on the horizontal angle of gaze in space.

### 2.13. Statistical analysis

When the conditions of usage have been checked (homogeneity of variances and normal distribution), analyzes of variance (Anova) with repeated measures have been performed in order to assess the effect of the trial (1–4), the visual condition (light, darkness), the trajectory type (limaçon, eight), and the considered body segment. A threshold of *p* < 0.05 has been considered significant. Newman-Keuls tests have been used for the *post-hoc* analysis whenever necessary. For non-normally distributed variables, Wilcoxon tests with Bonferroni corrections for multiple comparisons have been performed.

## 3. Results

### 3.1. Trajectory execution and kinematics

**Figure 2:**
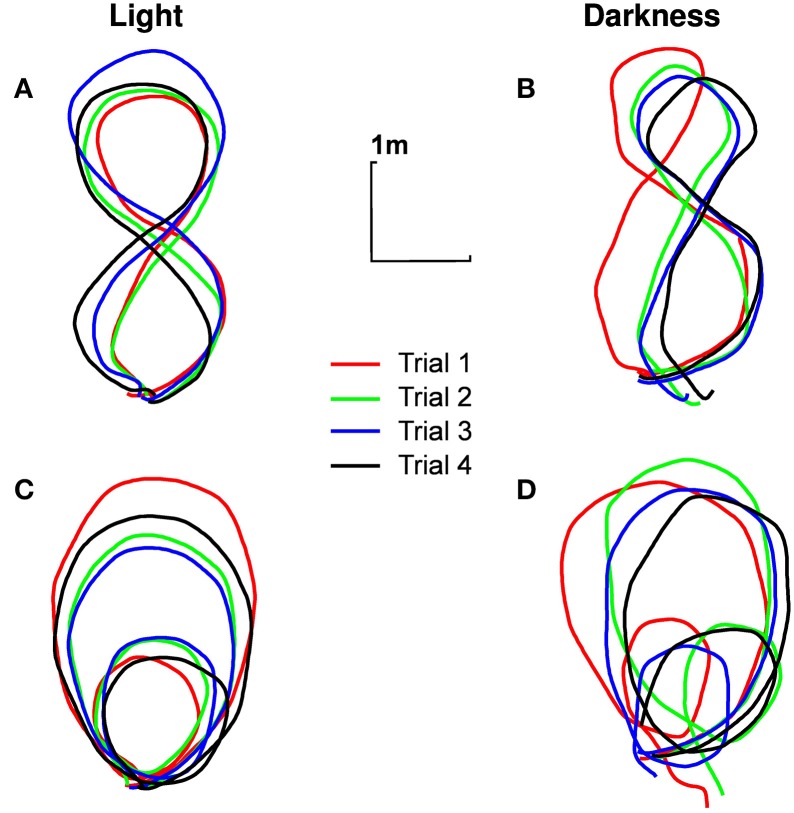
**Typical trajectories followed by participants**. Trajectory of the pelvis of participant n°1 in the ground plane, for different experimental conditions [**(A)** eight shape in light, **(B)** eight shape in darkness, **(C)** limaçon in light, **(D)** limaçon in darkness]. The four trials are presented (in order of realization: red, green, blue, black).

All the participants correctly performed the required trajectories, respecting all dimensions relevant to our study (i.e., importance of the global trajectory but no necessary precise looping to the starting point), for all trials. The darkness condition has been a little more difficult to realize though: the trajectories followed in darkness were obviously not as close to the ideal trajectory (shown on the sheet of paper) as the trajectories followed in light (Figure 2).

The instantaneous speed of the pelvis has been calculated for each trial of each participant. A Three-Ways repeated measures ANOVA [visual condition (2) × trajectory (2) × trials (4)] revealed a simple effect of the visual condition [*F*_(1, 9)_ = 54.12, *p* < 0.001, η^2^*p* = 0.85]. On average, the participants' speed is lower in darkness (0.67±0.10 m/s) than in light (0.80±0.14 m/s). Participants were also slower in the eight shape (0.76±0.11 m/s) than in the limaçon [0.71± 0.13 m/s, *F*_(1, 9)_ = 49.34, *p* < 0.001, η^2^*p* = 0.84] and slightly increased their locomotion speed between the first (0.70± 0.12 m/s) and the next three consecutive trials [0.74± 0.10 m/s, *F*_(3, 27)_ = 8.89, *p* < 0.001, η^2^*p* = 0.49].

### 3.2. Spatial anticipation of gaze and head compared to the trajectory

**Figure 3:**
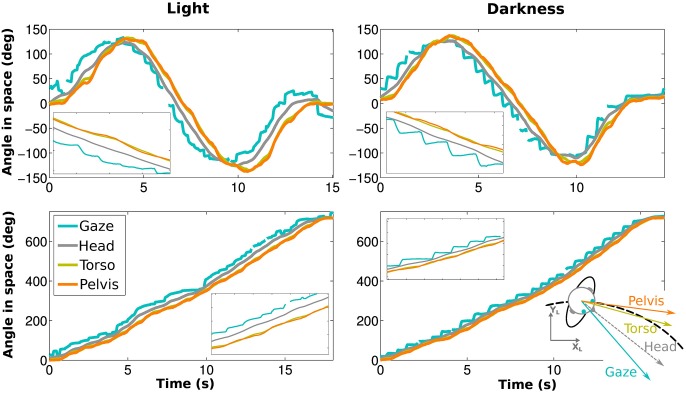
**Angle of gaze (blue), head (gray), torso (yellow) and pelvis (orange) while walking on an elongated eight shape (top) and a limaçon (bottom) for the same participant**. The left and right figures correspond to light and darkness conditions, respectively. Blowups (X5) are shown as well.

In both visual conditions, gaze has the most eccentric orientation, followed by the head, and then pelvis (Figure 3). We therefore confirmed the existence of gaze anticipation over the head direction which itself anticipates the trajectory direction. Moreover, the head precedes shoulders and torso, while shoulders and torso orientations are compatible (see Appendix Supplementary Results and Supplementary Figure 2).

### 3.3. Comparison of the anticipation properties

In order to compare the spatial anticipation in darkness and in the light, we computed for each trial the mean angles of the horizontal components of the segments (i.e., directions) of gaze and head with respect to the horizontal angle of the pelvis (see Appendix Supplementary Results for shoulder and torso). When this angle is positive, the considered segment is oriented toward the center of the curvature.

**Figure 4:**
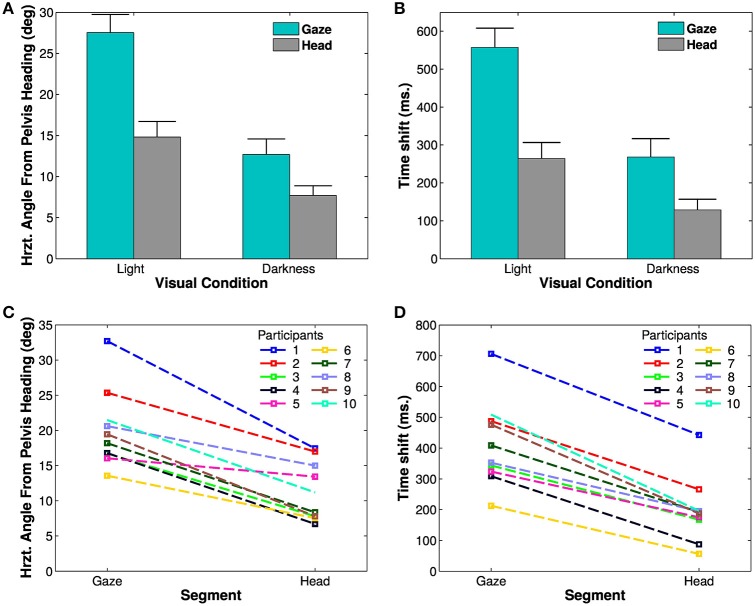
**Spatial and temporal anticipation of the body segments**. **(A,B)** Averaged anticipations in both visual conditions are represented (left: in light, right: in darkness). All differences are statistically significant (significance threshold: *p* < 0.05). The vertical bars represent the between-participant standard deviation. **(A)** Spatial anticipation of gaze (blue) and head (gray) with respect to the tangent to the pelvis orientation. **(B)** Time shift of the different body segments with respect to the pelvis movement. **(C,D)** Average spatial anticipation **(C)** and time shift **(D)** of the body segments for each participant. Each color corresponds to one participant.

A Four-Way ANOVA [segment (2) × visual condition (2) × trajectory (2) × trials (4)] revealed an effect of the visual condition [*F*_(1, 9)_ = 31.68, *p* < 0.01, η^2^*p* = 0.78, Figure 4A]. The spatial anticipation of the different segments is larger in light (21.16±6.18°) than in darkness (10.17±4.75°). The gaze (20.1±5.52°) is anticipating more than the head [11.23±4.18°, *F*_(1, 9)_ = 63.8, *p* < 0.01, η^2^*p* = 0.88]. The interaction between visual condition and segment factors is also significant [*F*_(1, 9)_ = 126.43, *p* < 0.01, η^2^*p* = 0.93]. *A posteriori* comparison indicated that gaze and head anticipations were significantly smaller in darkness (gaze: 12.67±6.05°; head: 7.67±3.79°) than in light (gaze: 27.53±6.91°; head: 14.79±6.02°). Both parameters are almost halved in the dark.

These results, i.e., less anticipation in darkness, anticipation of gaze over head, itself anticipating the trajectory (and the other body segments), are observable for all the participants (Figure 4C).

To determine the evolution of movement in the horizontal plane of the body segments, cross correlations have been calculated with respect to the pelvis horizontal angle (Bernardin et al., 2012). A Four-Way, repeated measures ANOVA has been performed on the lags from the cross correlations. The temporal anticipation (lag) of all segment is less important in light than in darkness [*F*_(1, 9)_ = 33.45, *p* < 0.001, η^2^*p* = 0.79]. A simple effect [*F*_(3, 27)_ = 74.32, *p* < 0.001, η^2^*p* = 0.89] of the considered segments is also observed. Gaze is the most anticipating segment compared to the pelvis (400±50 ms), followed by the head (200±10 ms). *A posteriori* comparisons show a significant difference between gaze and head.

An interaction between segments and visual conditions is also noticed [*F*_(3, 27)_ = 35.32, *p* < 0.01, η^2^*p* = 0.79, Figures 4B,D]. The anticipation of gaze and head is greater is light (gaze: 557.25±160 ms; head: 264.25±134 ms) than in darkness (gaze: 268.5 ±152 ms; head: 129 ± 88 ms). *A posteriori* comparisons highlight a significant difference between gaze and head segments across the visual conditions.

### 3.4. Independency of anticipation from walking speed and trajectory curvature

The characteristics of gaze and pelvis coordination (e.g., the magnitude of anticipation in space) could *a priori* vary according to both the walking speed and the curvature of the trajectories, as both factors could differ between light and darkness. Therefore, we analyzed to what extent the differences described between visual conditions can be explained by these variables before interpreting the statistical results. This analysis (presented in details in Appendix Supplementary Results) speaks in favor of an independency of the anticipation to the curvature and speed in our data. Therefore, the observed differences of anticipation are only due to the visual condition.

### 3.5. Head and gaze elevation

**Figure 5:**
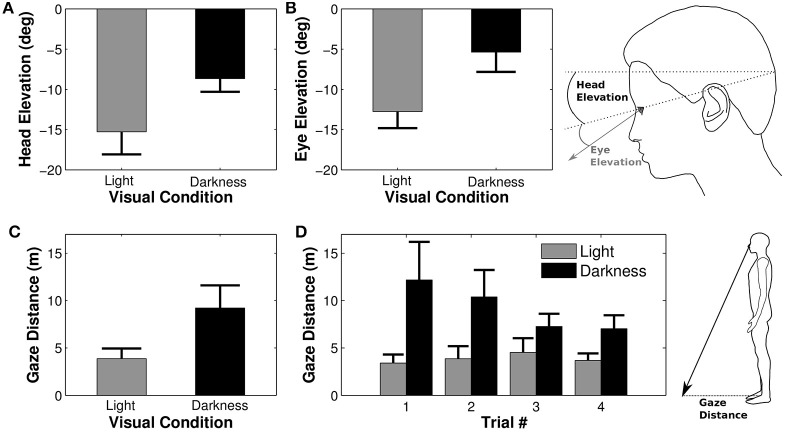
**Elevation of the eye and the head**. Average elevations of the head **(A)** and of the eye in the head **(B)** depending on the visual condition. Negative values indicate a downward orientation (below horizon for the head and below primary eye position for the eye). Large head and eye elevations lead to a shorter distance between gaze/floor intersection and the projection onto the ground of the right orbit [i.e., gaze distance, **(C)**, **(D)**].

Previous analyses focused on anticipations in the horizontal plane. However, head and gaze orientations were also modified in elevation (pitch). A Four-Way ANOVA revealed that the average head elevation was not different between trials [*F*_(3, 27)_ = 1.04, *p* = 0.39] or between trajectories [*F*_(1, 9)_ = 0.29, *p* = 0.49]. However, the head was more tilted downwards in light than in darkness [*F*_(1, 9)_ = 13.17, *p* = 0.005, η^2^*p* = 0.59, see Figure 5A]. The elevation of the eye in the orbit was similarly influenced by experimental conditions. Neither trial [*F*_(3, 27)_ = 0.19, *p* = 0.89] nor trajectory [*F*_(1, 9)_ = 0.03, *p* = 0.87] factors significantly affected the eye elevation, whereas the effect of the visual condition comes out clearly [*F*_(1, 9)_ = 17.36, *p* = 0.002, η^2^*p* = 0.66, see Figure 5B], meaning that the eye was more tilted downwards in light than in darkness.

The combined downward elevations of head in space and eye in head lead to a larger distance between the participant's position and its gaze projection onto the ground (gaze distance) in darkness condition as compared to the light condition [*F*_(1, 9)_ = 11.38, *p* = 0.008, η^2^*p* = 0.56, Figure 5C]. Gaze distance also significantly decreased between the first and the last trial [*F*_(3, 27)_ = 3.30, *p* = 0.035, η^2^*p* = 0.27, Newman-Keuls *p* < 0.05]. The analysis revealed an interaction between trial and visual condition factors [*F*_(3, 27)_ = 3.17, *p* = 0.04, η^2^*p* = 0.26, Figure 5D], indicating that gaze distance was significantly larger in the first trial as compared to all others in darkness.

### 3.6. Analyzing the nystagmus

#### 3.6.1. Existence of nystagmus

**Figure 6:**
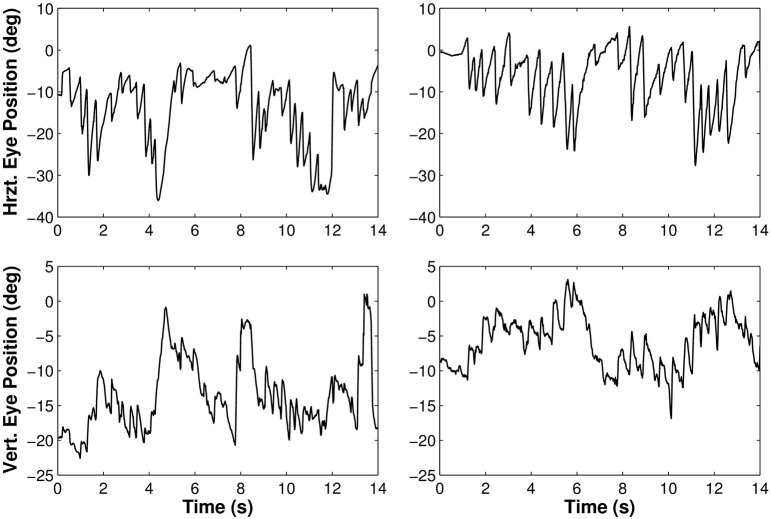
**Rotations of the eye in the orbit of one participant walking along the limaçon in light (left) and in darkness (right)**. The upper part of the figure corresponds to horizontal movements and the lower part to vertical movements.

Besides the analysis of the average gaze and head behaviors with respect to the pelvis trajectory, we observed the expected nystagmus in both visual conditions (Figure 6).

In order to pinpoint the functional aspect of anticipation, more advanced studies of ocular movements have been performed: (*i*) functional distinction and characterization of every movement observed (quick phases and reverse-saccades), (*ii*) gaze and visual stability (gain) during slow phases for the different nystagmi.

#### 3.6.2. Slow and quick phases basic characteristics

Slightly more quick phases are detected in darkness (2.23±0.42 Hz) than in light [1.89±0.44 Hz, *F*_(1, 9)_ = 43.55, *p* < 0.01]. The same feature is observed for slow phases [light: 2.11±0.46 Hz, darkness: 1.75±0.50 Hz, *F*_(1, 9)_ = 39.87, *p* < 0.01].

**Figure 7:**
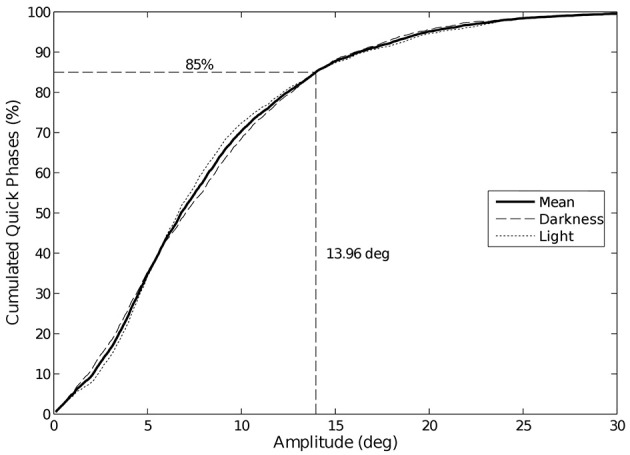
**Cumulative distribution of eye quick phases amplitude**. 85% of the quick phases have an amplitude lower than 14°. We did not observe any change in amplitude between visual conditions.

The visual condition impacts neither the amplitude nor the duration of the phases, whatever the trajectory is. Note that 85% of the quick phases have an amplitude lower than 14° (Figure 7), whatever the trajectory or visual condition is.

#### 3.6.3. Slow phases are more stable in the laboratory frame

In order to characterize the stability of the gaze during slow phases, we compared its horizontal rotational speed between an allocentric reference frame (Laboratory) and an egocentric reference frame (Head). The ANOVA analysis of angular speed during SP shows that the horizontal gaze speed in the allocentric reference frame is lower than the eye speed in the egocentric reference frame [*F*_(1, 9)_ = 49.63, *p* = 5.86×10^−5^, η^2^*p* = 0.85; in Lab.: 12.06±4.11°/s, in Head: 30.26±8.47°/s]. There is no effect of the visual condition [*F*_(1, 9)_ = 0.47, *p* = 0.51].

However, the SP speed is lower along the limaçon (16.28±7.38°/s) than along the eight shape [26.03±4.46°/s, *F*_(1, 9)_ = 19.66, *p* = 0.002, η^2^*p* = 0.68].

#### 3.6.4. Position of the eye in the head at beginning and end of the quick phases

**Figure 8:**
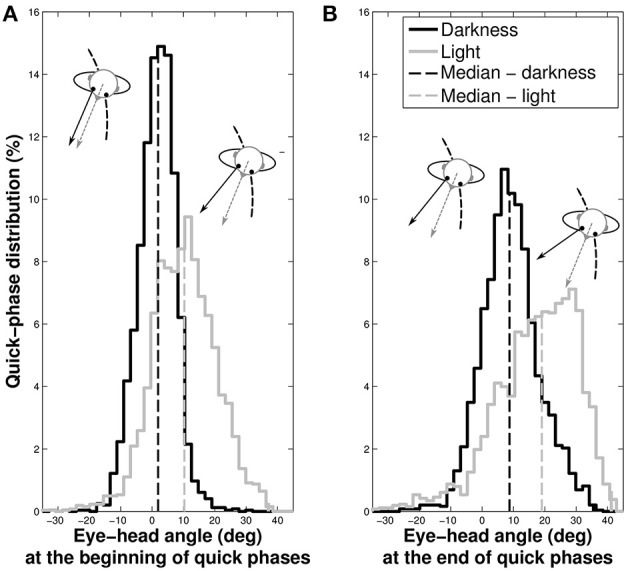
**Distributions of the eye position with respect to the head at the beginning (A) and at the end (B) of quick phases**. The gray spectrum represent data taken in light and the black spectrum the data in darkness. The dashed vertical lines indicate the median of the distributions.

We previously showed that gaze direction anticipated the head direction. To determine whether it is always the case during the entire slow phase, we analyzed the position of the eye with respect to the head at the beginning and the end of the quick phases (Figure 8).

In darkness, the eye is nearly at the center of the orbit at the beginning of the quick phases (median = 1.61±5.93°), while it is off-centered in light (median = 10.23±10.01°, Wilcoxon *p* < 0.001). Similarly, the median position of the eye at the end of the phase is closer to the center of the orbit in darkness (median = 9.19±9.04°) than in light, where it is off-centered (median = 17.34±13.01°, Wilcoxon *p* < 0.001).

These results mean that in both visual conditions, gaze anticipates the head direction during the whole nystagmus. A notable difference is that quick phases are elicited when the eye is in its primary position in darkness, and when it is in advance with respect to the head in light.

### 3.7. Reverse-saccades

We also performed an analysis of reverse-saccades, that mostly anticipate the inflection point of the elongated eight trajectory. The quick phases being always directed toward the heading direction, the reverse-saccades consequently correspond to quick phase eye movements in the opposite direction to the direction of displacement. Overall all conditions, 592 reverse-saccades have been counted (11.62% of the 5093 quick phases). The number of reverse-saccades per participant and trial (3.70±2.35) does not depend on the visual [*F*_(1, 9)_ = 1.45, *p* = 0.26] or the trajectory conditions [*F*_(1, 9)_ = 3.96, *p* = 0.08].

**Figure 9:**
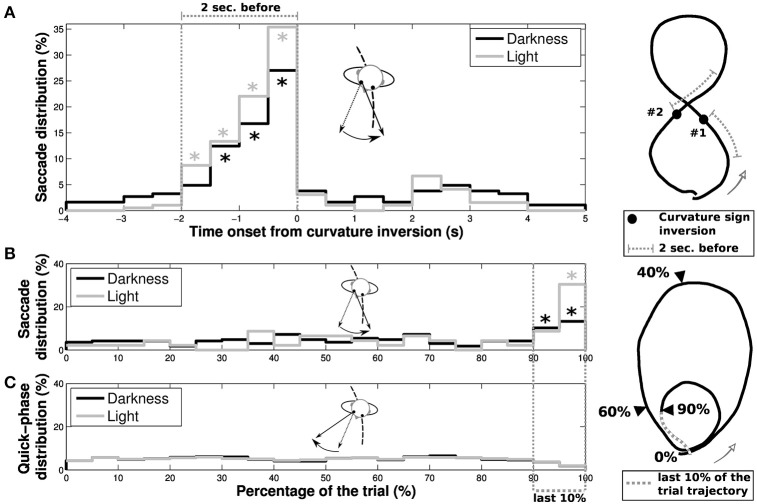
**Characteristics of reverse-saccades occurrence**. The reverse-saccade occurrence depends on different parameters for **(A)** elongated eight and **(B)** limaçon shapes. **(A)** Distributions of reverse-saccades as a function of the time onset from one of the two curvature sign inversion present in the extended height shape (black and gray for darkness and light conditions, respectively). A large proportion of reverse-saccades are elicited in a period of 2 s before the curvature sign inversion. Stars (^*^) indicate the bins with a significant difference (i.e., with a value higher than the statistical error $\sqrt{N}$). **(B)** Distribution of reverse-saccades occurrence as a function of the evolution of participant's progression on the overall limaçon shape. **(C)** The quick phases (without reverse-saccades) distribution as a function of the participant progression on the trajectory is also presented, for darkness (black) and light (gray) conditions. On the limaçon, a large proportion of reverse-saccades are elicited in the last 10% of the trajectory (39.13% and 23.49% of the reverse-saccades in light and darkness conditions, respectively). Stars (^*^) indicate the bins with a significant difference.

In order to determine the nature of these reverse-saccades, we focused our analysis on their occurrence both as a function of the progression on the trial (limaçon and eight shapes) and the time onset from an inversion in the curvature sign (only for eight shapes, see Figure 9). For eight shapes, a majority of reverse-saccades are elicited in the last 2 s before the curvature sign inversion (62.43% and 79.49% in the 2 s before inversion, to be compared to 9.94% and 5.13% in the 2 s after inversion, for darkness and light conditions, respectively). Moreover, if we only consider reverse-saccades out of this 2 s period before inversion, 59.89% (darkness) and 70.04% (light) of the remaining reverse-saccades were elicited in the last 10% of the trial. For limaçon shapes, 23.49% (darkness) and 39.13% (light) of the reverse-saccades were elicited in the last 10% of the trial.

### 3.8. Horizontal gains of the nystagmi slow phases

**Figure 10:**
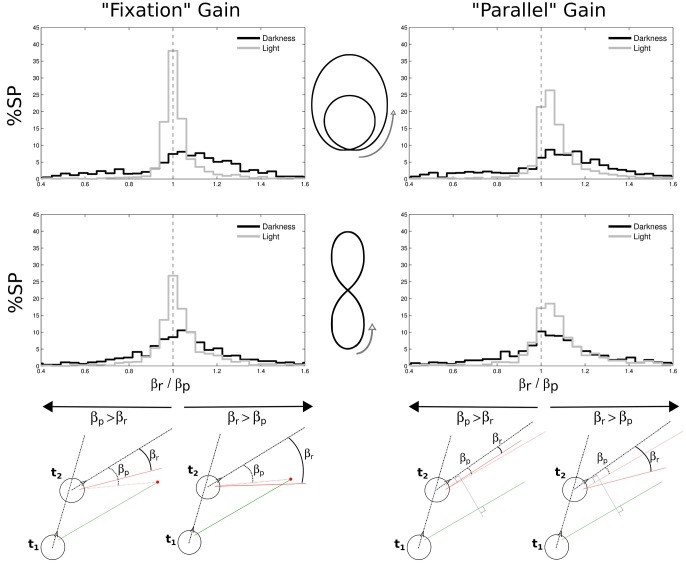
**Gains in space of nystagmi slow phases**. Two different gains were considered, both in the horizontal plane: a “fixation” gain corresponding to the tendency of gaze during a slow phase to stay aligned with a location on the ground (left part of the figure), and a “parallel” gain assessing the stability of gaze direction in the horizontal plane (right part of the figure). Both gains were computed from a ratio between the real gaze direction (β_*r*_, from the head direction) and a predicted gaze direction (β_*p*_, from the head direction). The prediction could either be to consider gaze directed toward a point on the ground determined from the previous experimental gaze direction (bottom left) or to consider a gaze parallel to its previous direction (bottom right). A close-to-one gain means that the predicted and real angles were close to each other, as a gain larger than one means that the real angle is wider than the predicted angle. These gains were computed for limaçon (top) and elongated eight (middle) shapes, and separately in darkness (black) and in light (gray). Green and red segments represent gaze direction at the beginning and at the end of a slow phase, respectively.

In order to characterize the nature of the slow phases and better understand their function, we defined two measures of their possible gain in the horizontal plane. The first definition is “fixation gain,” measuring the tendency of gaze to stay aligned with a location on the ground, and the second definition is “parallel gain,” aiming at assessing the stability of gaze parallel direction in the horizontal plane (see Figure 10, bottom).

To compute these gains, we considered, at two successive moments *t*_1_ and *t*_2_ of a single slow phase, the two following horizontal angles: the angle β_*r*_ (*r* for real) between the gaze direction at *t*_2_ (continuous red line) and the head direction at the same moment (dashed black line), and the angle β_*p*_ (*p* for predictive) between the head direction and the direction that gaze would have at *t*_2_ if it was either directed toward the same target than the gaze at *t*_1_ (fixation case, Figure 10, bottom left), or if it was parallel to the gaze at *t*_1_ (parallel case, Figure 10, bottom right), respectively. The gain μ was computed every two frames (i.e., at 25 Hz) and then averaged over for each slow phase, as follows:

(2) $$\mu =\frac{\sum_{i\;=\text{ onset}}^{\text{offset}}\left(\beta_{ri}/\beta_{pi}\right)}{\text{offset}-\text{onset}}$$

We found that the medians of both gains are close to one. However, the distributions of both gains for all trajectories are more spread out in darkness than in light (all Levenne *p* < 0.001; σ = 0.43 and σ = 0.25 in darkness and light, respectively).

Another interesting result is that in light, *fixation gains* are slightly but significantly closer to unity (medians 1.0059 for limaçon and 1.0139 for elongated eight) than *parallel gains* (both Wilcoxon *p* < 0.01; medians 1.0466 for limaçon and 1.0569 for elongated eight). This indicates that in light, the horizontal angle in space during slow phases of nystagmus tends to increase (because *parallel gain* is > 1) and globally stabilizes gaze direction around a focalized location on the ground plane. In darkness, no significant difference is observed between both kinds of gain (Wilcoxon *p* > 0.05). However, 65% of the parallel gains distribution is above unity, meaning that the horizontal gaze angle progresses in space during slow phases also in darkness more than it regresses.

The variability of gains in the dark is higher than in the light.

### 3.9. Other attempts to determine the nature of anticipating behaviors in the dark

To try to further characterize the difference of gaze (and head) anticipations across the two lighting conditions, we conjectured that the anticipation in the dark was a residual of the well-known anticipation in light. It thus could be the result of two different mechanisms: the residual gaze anticipation in darkness would either correspond to the subtraction of an angular shift present in light, both for the gaze and for the head; or it would match a weakening of anticipation and should be better modeled by a division of the anticipatory angles by a certain coefficient.

The results are shown in the Appendix Supplementary Results. For the gaze anticipation, the performance of the two models is almost identical, while for the head the score of the divisive model is a little better than for the subtractive model. However, the two models seem overall comparable. Thus, we concluded both models can explain the data, and were not able to discriminate between the two.

## 4. Discussion

### 4.1. Anticipation of the locomotor trajectory by gaze and head is maintained without vision

Our results confirmed that during human free locomotion on curved paths, the horizontal component of gaze anticipates the horizontal component of head direction which itself anticipates the trajectory orientation. This holds both in the light and in total darkness. Thus, we confirmed that the hypothesis of a visual function is not sufficient to explain gaze and head anticipations during locomotion.

This article is the first to report this behavior in the dark for humans along imagined complex trajectories with varying curvature; it was known before for human locomotion in the dark around a 90° corner with obstacle (a 50 cm high and wide box, Grasso et al. 1998) and for trained monkeys locomotion on a circular path (Solomon and Cohen, 1992a,b). It was also known for human locomotion on complex path in the light (Bernardin et al., 2012).

Concerning the head, Courtine and Schieppati (2003a) reported anticipation in the dark, without obstacle on circular trajectories, but in their experiment the lights-off condition followed the lights-on condition, which can be considered as a kind of learning; moreover (Courtine and Schieppati, 2003a) made no analysis of gaze movements. With respect to Grasso et al. (1998), the novelty of our experiment consists in the greater complexity of the trajectory, with curvature variation, and the absence of obstacle, either seen or memorized. For instance, the head and gaze movements reported in Grasso et al. (1998) could have been due to the attention directed toward the obstacle in the corner. In Grasso et al. (1998), eyes were closed, and eye movements were recorded by electrooculography. In our experiment, we used a more precise technology (i.e., better spatial resolution, reduced drift and 2D eye-tracking), eyes were open, and eye movements were recorded by a head-mounted video eye-tracker (50 Hz). Moreover, we considered a sufficiently large number of subjects, trials and conditions to allow for a rigorous statistical analysis. In contrast with (Solomon and Cohen, 1992a,b), our subjects were humans, our trajectories were less constrained and comprised curvature changes, allowing to test that both head and gaze anticipate the variations of curvature. Moreover, some characteristics of the anticipation that we observed in humans and for complex curves are not similar to the ones that Solomon and Cohen observed for monkeys on learned circular paths.

One property of gaze is particularly remarkable and was never reported before: during a locomotor trajectory that turns continuously to the same side, i.e., without inflection point, the horizontal component of gaze is purely monotonic in a fixed room frame, which means that it is always either increasing or decreasing. This horizontal component therefore predicts the trajectory orientation without redundancy. This remains true even without vision and in spite of the presence of nystagmus (see Figures 3, 4).

### 4.2. Comparing anticipations in light or darkness

The most notable difference we observed is a reduction of horizontal gaze and head anticipation by a half in darkness. In other words, though remaining in absence of vision, anticipation is increased by vision. Indeed, head and gaze anticipations are diminished in the dark, both in space (angular amplitude) and time (measured as the time shift). This observation is compatible with the results of Courtine and Schieppati (2003a) who also observed less changes in heading in the dark as compared to a free-vision situation.

The most notable similarity between the observed anticipation in the light and in the dark, is the existence of a clear horizontal eye nystagmus (alternation of quick and slow phases of the eye direction with respect to the head), with many key characteristics in common across the two vision conditions. This is a highly non-trivial result, because it may seem, at first sight, in contradiction with the diminution of the anticipation in the dark. This new result could be used to deduce new insight in the fundamental process of locomotion planning by gaze.

Though the nystagmus frequency may be slightly higher without vision, the mean amplitude of the quick and slow phases of the eye nystagmus are similar in both conditions. Yet, the standard deviation of the quick phases distributions is smaller in the dark (9.04°) than in plain light (13.01°). The eye quick phase amplitude and duration were not affected by the trajectory condition either [85% of quick phases amplitudes fall under 14°, as commonly observed by Bahill et al. (1975)].

However, an important difference between light and dark conditions appears at the beginning and at the end of the quick phases of the nystagmus. Without vision, the quick phases start when aligned with the head direction (i.e., whereto the nose is pointing) and stop likewise, while with vision we measure an offset angle which shifts the gaze out and in advance over the head. We interpret this observation as the existence, in the lights-on condition, of a “residual angular anticipatory shift” which is not present when tested in the dark.

These two results—a lesser anticipation by gaze and head in the dark, and the existence of a residual gaze shift in light—are new.

In both vision conditions, the velocity of the slow phases of the nystagmus was positive, thus we cannot confirm here the exact definition of travel fixations (Patla and Vickers, 2003). When computing the gains under the assumption of local fixations or of a parallel gaze, we found in both cases a rather good gain in light (close to 1) and a larger gain (>1) in darkness. Without visual information, gaze is drifting away, as if it was caught by intermediary—though invisible—cues in space, as confirmed by the smaller gaze elevation without vision. In contrast, in the vision condition, the gaze direction in slow phases is consistent with a visual stabilization onto a fixed target on the ground and with a stabilization of the local optical flow (Lappe et al., 1998; Authié and Mestre, 2011) using optokinetic and pursuit mechanisms.

One can also note that the horizontal gains are more dispersed (deviated from the mean) in the dark. This may be simply due to the absence of visual information in the dark, but this can also be explained by a functional difference, or in other words, by a horizontal gaze drift in the dark. And as for the vertical drift, it could provide some information about the position and assist the simulation of the forthcoming trajectory.

These observation tend to confirm the hypotheses that we exposed in the introduction.

### 4.3. Predictive nystagmus during locomotion

It had been already noted (Solomon and Cohen, 1992b; Grasso et al., 1998) that walking elicits an eye nystagmus (in humans and primates, notably) and that the quick phases of this nystagmus drag gaze toward the inner edge of the bend in a curved trajectory (see also Authié and Mestre, 2011; Lappi et al., 2013; in driving tasks). However, no previous work had precisely described this function of nystagmus during walking in humans.

Solomon and Cohen (1992a,b) were the first to describe quantitatively the eye and head nystagmus during locomotion, in light and darkness, for one rhesus and one cynomolgus monkey. However, they spoke of “compensatory gaze nystagmus” and do not noticed the fact that this nystagmus supports the prediction by the gaze of the future body orientation. As mentioned in the introduction, an examination of their recordings of eye and head movements confirms this predictive character. The nystagmus frequency that they measured is comparable to ours, and about 2 Hz. But major differences distinguish their observations on monkeys from our observations on humans: the nystagmus has a higher frequency in light for monkeys, where the contrary holds for humans; larger amplitudes of quick phases appear in darkness for monkeys without notable difference in speed, which is opposite to what we found for humans.

The physiology of quick phase eye movements is known to be flexible and modular (McCrea et al., 1980), in particular in a natural situation, adapted to vestibular and/or optokinetic signals. Our results argue in favor of the existence of a single anticipatory and stabilizing mechanism present with and without vision, even if it mixes several sources in various proportions. However, this common mechanism presents distinct characteristics with or without vision, which seems to indicate diverging functions.

### 4.4. The differences of gaze anticipation between light and dark conditions could be due to the ground of beating field shift which appears with vision

Let us define the *beating field* (BF) of nystagmus as the angular range of the eye with respect to the nose orientation during a quick phase, between its beginning and its end (Meier and Dieringer, 1993; Siegler et al., 1998). The variation of the upper bound of the BF during the nystagmus is named the *shift* of BF. If the BF amplitude is constant over the nystagmus, this shift is also the variation of the lower bound of BF.

We observed that both head and gaze have a constant residual precession angle in the curved direction. This implies the existence of a global shift of BF when going from darkness to light.

Our results indicate that this global shift could explain all main differences between gaze anticipation in the light and in the dark.

First we argued that the variability in the trajectory execution (curvature and speed) in the dark is not responsible for the reduction of the anticipation. Then we assumed that the difference of anticipation might either be a mere subtraction of the residual horizontal angles of gaze in space with respect to the trajectory orientation, or that the anticipation was only weakened in darkness. We found out that either model would be sufficient to explain most of the differences in the eye movements between the two conditions, and that the “divisive” model would perform slightly better to report the differences in head movements with respect to the trajectory. The fact that the eyes come back to the sagittal plane in the dark and that the nystagmus kinematics are unchanged might be in favor of the “subtractive” model for the eyes, even if a moderately higher frequency of the nystagmus in the dark could be evidence of its vestibular origin (McCrea et al., 1980).

Therefore, we suggest that the main differences between the two conditions originate from one factor, that is a shift of the BF of the eye and the head in the direction of trajectory curvature in light.

### 4.5. What is the role of gaze and head anticipation?

Anticipation of locomotion by gaze and head assists the brain in several tasks: (1) exploit visual information, for instance through fixations, pursuit, and optic flow stabilization; (2) exploit non-visual information, such as vestibular and proprioceptive stimuli, perhaps modulated by the oculomotor activity; (3) combine both sources of information with the help of cognitive mechanisms, as imagination or mental simulation, in order to optimize future motion and space perception.

The specific trace of visual information flow is the permanent ground of shift of the BF, giving a residual anticipation of gaze with respect to the head, and of the head with respect to the trajectory. The specific trace of an online motor predictive control is the laps of time between gaze and body orientation, between 100 ms and 300 ms, which is compatible with self-motion preparation and reaction.

The exact way in which the brain integrates the anticipative gaze signals to subserve this motor function has to be further precised, but we could suggest that gaze an head anticipation help stabilizing the brain sensors, visual, vestibular and proprioceptive ones, and according them together, provides the best conditions for extracting dynamical invariants of self-motion, controlling equilibrium, and constructing coherent spatio-temporal perception.

Based on what precedes, we suggest that a fundamental function of gaze is to represent self motion and to stabilize the perception of space during locomotion, regardless of the vision condition. Another compatible and important function of gaze is to simulate the future trajectory.

Reed-Jones et al. (2009) demonstrated the importance of the orientation of the eyes in a direction change by constraining eye movements. They show that the postural anticipatory behavior disappears when entering the bend whenever gaze is constrained.

Cinelli and Warren (2012) studied the effects of different intentions and constraints on the head anticipation in rotations. They established that anticipatory movements of the head are neither necessary nor sufficient to initiate a change of direction in human locomotion, when a visual locomotor goal is present. However, our present research show that, under nearly natural conditions, even in the dark, human subjects spontaneously anticipate the trajectory with their gaze. It is likely that the absence of coupling between gaze and head anticipation and steering control observed by Cinelli and Warren (2012) was due to the resort to another steering mechanism or a higher level of attention. We could have varied the mode of presentation of the trajectory (with normal vision) and measure the potential disturbances of the kinematics of locomotor trajectories (e.g., minimum variance, minimum jerk, trajectory geometry). As a second step, it would be interesting to compare these results with the disturbances obtained without vision and without head and eye movements. Are these disturbances more important when vision is denied? Even so, we do not state that the anticipation of the eyes and head is a necessary condition to locomotion. Steering does not require gaze anticipation (Cinelli and Warren, 2012), as it is possible to walk toward a target without staring at it. But when participants reproduce a trajectory, without a visible locomotor goal, this anticipation is spontaneously present. We support the hypothesis that this spontaneous behavior reflects several functional components of gaze: representing self motion, stabilizing the perception of space during locomotion, and simulating the future trajectory, regardless of the vision condition. However, we cannot exclude the hypothesis that other strategies, as target-heading angle strategy (Fajen and Warren, 2003) could be used to control steering during locomotion. This question deserves further investigations in future studies.

### 4.6. Gaze behavior during changes of trajectory curvature: “reversal of saccades”

Aside the standard anticipation of gaze and head that results from nystagmus and fixations, we observed saccades, that we could interpret as anticipation of new segments in the future trajectory, apparently more cognitive than online motor anticipation: just before an inflection point of the trajectory the gaze tends to point in the direction of the future curvature, which generates a saccadic movement of gaze in the reverse direction, named a *reverse-saccade*. Our results prove that these reverse-saccades have the same statistical properties for the two visual conditions.

From this we suggest that the prospective function, to imagine the future part of the global trajectory, is not meant to gather purely visual information, but also contributes to an internal preparation of the motor execution or to the mental simulation of the future trajectory.

We observed that 10% of the eye quick phases were reverse-saccades. These reverse-saccades are comparable across visual conditions (begins −2 s in light and −1.5 s in darkness) and support gaze anticipation. These gaze shifts could reflect the trajectory segmentation in the short-term planning process, as previously described in hand movement segmentation around an inflection point of the trajectory (Viviani and Terzuolo, 1982). Indeed, inflections in the trajectory have a stereotypical velocity profile (Bennequin et al., 2009) that can be reflected in special gaze behavior.

We suggest that the sudden reversal of the gaze could also be due to the well-known fact that the neural centers, such as the superior colliculus and the frontal eye fields, involved in the generation of rapid eye movements (saccades, quick phases of nystagmus) are lateralized (Yoshida et al., 1982; Green and Angelaki, 2003; Wei and Angelaki, 2004). Therefore, when the curvature of the trajectory changes sign, i.e., at inflection points, the control switches from one generator to the other.

### 4.7. Remarks on the neural basis of gaze anticipation

The control of gaze movements by the brain, in humans as in other vertebrates, is based on fundamental subcortical networks, in the brainstem, pontine nuclei, tectum or colliculus, and optic tracts, modulated by the activity of several cerebellar nuclei and vermis subdivisions. It also involves a large cortical network, with frontal, cingulate, parietal, and temporal components (Cf. Pierrot-deseilligny et al., 2004 for a review). These interacting networks produce eye and head movements that optimize stability in visual and vestibular perceptions, together with motor control. These eye movements combine saccades, pursuit, vergence and VOR, with optokinetic nystagmus (OKN) and vestibular nystagmus reflex (VNR, Cf. Solomon and Cohen, 1992a,b). During locomotion, in light and in darkness, we observed that the quick phases of nystagmus happen before the eyes come back in the sagittal plane, allowing gaze anticipation. For the two sorts of nystagmi, it was already observed in several conditions (unrelated to locomotion), that the quick phase is shifted during the nystagmus sequence to anticipate the movement of the head (cf. Meier and Dieringer, 1993; Siegler et al., 1998), the above mentioned phenomenon named “the beating field shift” (BFS). Several attempts were made to identify the neural bases of BFS, looking specifically at frontal or parietal eye field lesions, without much success (Bähring et al., 1994; Rivaud et al., 1994; Dieterich et al., 2003, 2009). In darkness, we observed that the quick phase was elicited when the eye came back in the sagittal plane. The precise control of this behavior could be achieved by the known networks, because it is known that eye movement control is lateralized. For instance in the brainstem, there is a subsystem generating quick phases to the right and another one generating quick phases to the left (Cf. Yoshida et al., 1982; Green and Angelaki, 2003; Wei and Angelaki, 2004). Moreover, in the parieto-frontal system, Vallar et al. (1999) have identified a representation of the sagittal plane of the body, which could provide the angle-related triggering information of the quick phase. There is also a fundamental bilateral symmetry in the cross inhibition of superior colliculus, which is the principal subcortical structure involved in orienting movements.

### 4.8. Evolution could have selected and adapted the nystagmus for locomotion

As underlined by many specialists, eye movements should be studied under ecological conditions (*cf*. Steinman, 2003). We argue that the nystagmus mechanisms can be studied under artificial conditions, while the nystagmus function cannot. Likewise, saccades functions can only be deduced from natural situations. For instance, in natural situations saccades are made by eyes and head together. The reverse saccades we observed could be part of motion representation and planning. Similarly, the nystagmus is known as a dynamic process compensating for the optic flow and for the movements of the head and eyes during locomotion or other natural movements. However, the characteristics of nystagmus that we observed with or without vision, indicate a common function for the nystagmus, whatever it is, vestibular or optokinetic, related to motor control or space perception or trajectory simulation, in proportions to be precised.

We finally suggest that the function of nystagmus dedicated to locomotion is very ancient and can have oriented the selection of nystagmus mechanisms during evolution.

## 5. Conclusion

To sum up, the whole set of our results demonstrates that gaze has a predictive function on the future displacement, regardless of the visual condition. This function is fulfilled in great part by a form of eye nystagmus, maybe of vestibular and proprioceptive origins in darkness, and mixing optokinetic, pursuit, and vestibular sources in light. The eyes nystagmus persists in darkness with the same amplitude of quick phases and almost the same frequency, and the main difference between light and dark conditions is the existence of a permanent background shift of BF toward the curvature of the trajectory in the light.

Gaze anticipation could take part in a modulation of the vestibular and proprioceptive input signals, possibly depending on the efferent copy of motor commands. This predictive function of gaze could serve many purposes: controlling the stability of motion, modulating the sensorimotor inputs, contributing to spatial updating and “mental simulation” of the future movement. Furthermore, gaze could belong to an anticipative frame that maintains and prepares space perception.

### Conflict of interest statement

The authors declare that the research was conducted in the absence of any commercial or financial relationships that could be construed as a potential conflict of interest.

## References

[B1] Authié C. N. Mestre D. R. (2011). Optokinetic nystagmus is elicited by curvilinear optic flow during high speed curve driving. Vision Res. 51, 1791–1800. 10.1016/j.visres.2011.06.010 21704061

[B2] Authié C. N. Mestre D. R. (2012). Path curvature discrimination: dependence on gaze direction and optical flow speed. PLoS ONE 7:e31479. 10.1371/journal.pone.0031479 22393363 PMC3290598

[B3] Bähring R. Meier R. Dieringer N. (1994). Unilateral ablation of the frontal eye field of the rat affects the beating field of ocular nystagmus. Exp. Brain Res. 98, 391–400. 8056062 10.1007/BF00233977

[B4] Bahill A. Adler D. Stark L. (1975). Most naturally occurring human saccades have magnitudes of 15 degrees or less. Invest. Ophthalmol. 14, 468–469. 1132942

[B5] Bennequin D. Fuchs R. Berthoz A. Flash T. (2009). Movement timing and invariance arise from several geometries. PLoS Comput. Biol. 5:e1000426. 10.1371/journal.pcbi.1000426 19593380 PMC2702097

[B6] Bernardin D. Kadone H. Bennequin D. Sugar T. Zaoui M. Berthoz A. (2012). Gaze anticipation during human locomotion. Exp. Brain Res. 223, 65–78. 10.1007/s00221-012-3241-2 22968738

[B7] Berthoz A. (1997). Le Sens du Mouvement. Paris: Odile Jacob.

[B8] Cinelli M. Warren W. (2012). Do walkers follow their heads? Investigating the role of head rotation in locomotor control. Exp. Brain Res. 219, 1–16. 10.1007/s00221-012-3077-9 22466410 PMC3592975

[B9] Courtine G. Schieppati M. (2003a). Human walking along a curved path. I. Body trajectory, segment orientation and the effect of vision. Eur. J. Neurosci. 18, 177–190. 10.1046/j.1460-9568.2003.02736.x 12859351

[B9b] Courtine G. Schieppati M. (2003b). Human walking along a curved path. II. Gait features and EMG patterns. Eur. J. Neurosci. 18, 191–205. 10.1046/j.1460-9568.2003.02737.x 12859352

[B11] Dieterich M. Bense S. Stephan T. Yousry T. A. Brandt T. (2003). fMRI signal increases and decreases in cortical areas during small-field optokinetic stimulation and central fixation. Exp. Brain Res. 148, 117–127. 10.1007/s00221-002-1267-6 12478402

[B12] Dieterich M. Müller-Schunk S. Stephan T. Bense S. Seelos K. Yousry T. A. (2009). Functional magnetic resonance imaging activations of cortical eye fields during saccades, smooth pursuit, and optokinetic nystagmus. Ann. N. Y. Acad. Sci. 1164, 282–292. 10.1111/j.1749-6632.2008.03718.x 19645913

[B13] Fajen B. R. Warren W. H. (2003). Behavioral dynamics of steering, obstable avoidance, and route selection. J. Exp. Psychol. 29, 343–362. 10.1037/0096-1523.29.2.343 12760620

[B14] Flanders M. Daghestani L. Berthoz A. (1999). Reaching beyond reach. Exp. Brain Res. 126, 19–30. 10333004 10.1007/s002210050713

[B15] Glasauer S. Amorim M.-A. Viaud-Delmon I. Berthoz A. (2002). Differential effects of labyrinthine dysfunction on distance and direction during blindfolded walking of a triangular path. Exp. Brain Res. 145, 489–497. 10.1007/s00221-002-1146-1 12172660

[B16] Grasso R. Glasauer S. Takei Y. Berthoz A. (1996). The predictive brain: anticipatory control of head direction for the steering of locomotion. Neuroreport 7, 1170–1174. 8817526

[B17] Grasso R. Prévost P. Ivanenko Y. P. Berthoz A. (1998). Eye-head coordination for the steering of locomotion in humans: an anticipatory synergy. Neurosci. Lett. 253, 115–118. 9774163 10.1016/s0304-3940(98)00625-9

[B18] Green A. M. Angelaki D. E. (2003). Resolution of sensory ambiguities for gaze stabilization requires a second neural integrator. J. Neurosci. 23, 9265–9275. 14561853 10.1523/JNEUROSCI.23-28-09265.2003 PMC6740579

[B19] Hicheur H. Berthoz A. (2005). How do humans turn? Head and body movements for the steering of locomotion, in 5th IEEE-RAS International Conference on Humanoid Robots (Tsukuba: IEEE), 265–270.

[B20] Hicheur H. Glasauer S. Vieilledent S. Berthoz A. (2005). Head direction control during active locomotion in humans, in Head Direction Cells and the Neural Mechanisms of Spatial Orientation, eds Wiener S. I. Taube J. S. (Cambridge, MA: MIT Press), 383–408.

[B21] Higuchi T. (2013). Visuomotor control of human adaptive locomotion: understanding the anticipatory nature. Front. Psychol. 4:277. 10.3389/fpsyg.2013.00277 23720647 PMC3655271

[B22] Hollands M. A. Patla A. E. Vickers J. N. (2002). “Look where you're going!”: gaze behaviour associated with maintaining and changing the direction of locomotion. Exp. Brain Res. 143, 221–230. 10.1007/s00221-001-0983-7 11880898

[B23] Imai T. Moore S. T. Raphan T. Cohen B. (2001). Interaction of the body, head, and eyes during walking and turning. Exp. Brain Res. 136, 1–18. 10.1007/s002210000533 11204402

[B24] Jeannerod M. (1988). The Neural and Behavioural Organization of Goal-Directed Movements. New York, NY: Clarendon Press/Oxford University Press.

[B25] Johansson R. S. Westling G. Bäckström A. Flanagan J. R. (2001). Eye-hand coordination in object manipulation. J. Neurosci. 21, 6917–6932. 11517279 10.1523/JNEUROSCI.21-17-06917.2001 PMC6763066

[B26] Kadone H. Bernardin D. Bennequin D. Berthoz A. (2010). Gaze anticipation during human locomotion A top-down organization that may invert the concept of locomotion in humanoid robots, in IEEE Conference on RO-MAN (Viareggio), 552–557.

[B27] Kaminiarz A. Königs K. Bremmer F. (2009). The main sequence of human optokinetic afternystagmus (OKAN). J. Neurophysiol. 101, 2889–2897. 10.1152/jn.00114.2009 19297517

[B28] Land M. F. Lee D. N. (1994). Where we look when we steer. Nature 369, 742–744. 8008066 10.1038/369742a0

[B29] Land M. Mennie N. Rusted J. (1999). The roles of vision and eye movements in the control of activities of daily living. Perception 28, 1311–1328. 10755142 10.1068/p2935

[B30] Lappe M. Pekel M. Hoffmann K. P. (1998). Optokinetic eye movements elicited by radial optic flow in the macaque monkey. J. Neurophysiol. 79, 1461–1480. 9497425 10.1152/jn.1998.79.3.1461

[B31] Lappi O. Pekkanen J. Itkonen T. H. (2013). Pursuit eye-movements in curve driving differentiate between future path and tangent point models. PLoS ONE 8:e68326. 10.1371/journal.pone.0068326 23894300 PMC3718775

[B32] Marigold D. S. Patla A. E. (2007). Gaze fixation patterns for negotiating complex ground terrain. Neuroscience 144, 302–313. 10.1016/j.neuroscience.2006.09.006 17055177

[B33] McCrea R. Yoshida K. Berthoz A. Baker R. (1980). Eye movement related activity and morphology of second order vestibular neurons terminating in the cat abducens nucleus. Exp. Brain Res. 40, 468–473. 7439286 10.1007/BF00236156

[B34] Meier R. Dieringer N. (1993). The role of compensatory eye and head movements in the rat for image stabilization and gaze orientation. Exp. Brain Res. 96, 54–64. 8243583 10.1007/BF00230438

[B35] Mestre D. R. Authié C. N. (2012). Why do we move our head during curve driving?, in Advances in Human Aspects of Road and Rail Transportation, ed Stanton N. A. (Boca Raton, FL: CRC Press edition), 412–419.

[B36] Niemann T. Lappe M. Büscher A. Hoffmann K. P. (1999). Ocular responses to radial optic flow and single accelerated targets in humans. Vision Res. 39, 1359–1371. 10343848 10.1016/s0042-6989(98)00236-3

[B37] Paillard J. (1982). The contribution of peripheral and central vision to visually guided reaching, in Analysis of Visual Behavior, Ingle D. J. Goodale M. A. Mansfield R. J. W. (Cambridge, MA: MIT Press), 367–385.

[B38] Patla A. E. Vickers J. N. (1997). Where and when do we look as we approach and step over an obstacle in the travel path? Neuroreport 8, 3661–3665. 9427347 10.1097/00001756-199712010-00002

[B39] Patla A. E. Vickers J. N. (2003). How far ahead do we look when required to step on specific locations in the travel path during locomotion? Exp. Brain Res. 148, 133–138. 10.1007/s00221-002-1246-y 12478404

[B40] Pierrot-deseilligny C. Milea D. Müri R. M. (2004). Eye movement control by the cerebral cortex. Curr. Opin. Neurol. 17, 17–25. 10.1097/00019052-200402000-00005 15090873

[B41] Pozzo T. Berthoz A. Lefort L. (1990). Head stabilization during various locomotor tasks in humans. Exp. Brain Res. 82, 97–106. 2257917 10.1007/BF00230842

[B42] Reed-Jones R. Reed-Jones J. Vallis L. A. Hollands M. (2009). The effects of constraining eye movements on visually evoked steering responses during walking in a virtual environment. Exp. Brain Res. 197, 357–367. 10.1007/s00221-009-1923-1 19582438

[B43] Reuschel J. Rösler F. Henriques D. Y. P. Fiehler K. (2012). Spatial updating depends on gaze direction even after loss of vision. J. Neurosci. 32, 2422–2429. 10.1523/JNEUROSCI.2714-11.2012 22396416 PMC6621798

[B44] Rivaud S. Müri R. M. Gaymard B. Vermersch A. I. Pierrot-Deseilligny C. (1994). Eye movement disorders after frontal eye field lesions in humans. Exp. Brain Res. 102, 110–120. 7895787 10.1007/BF00232443

[B45] Shepherd S. V. (2010). Following gaze: gaze-following behavior as a window into social cognition. Front. Integr. Neurosci. 4:5. 10.3389/fnint.2010.00005 20428494 PMC2859805

[B46] Siegler I. Israël I. Berthoz A. (1998). Shift of the beating field of vestibular nystagmus: an orientation strategy? Neurosci. Lett. 254, 93–96. 9779928 10.1016/s0304-3940(98)00671-5

[B47] Solomon D. Cohen B. (1992a). Stabilization of gaze during circular locomotion in darkness. II. Contribution of velocity storage to compensatory eye and head nystagmus in the running monkey. J. Neurophysiol. 67, 1158–1170. 1597705 10.1152/jn.1992.67.5.1158

[B48] Solomon D. Cohen B. (1992b). Stabilization of gaze during circular locomotion in light. I. Compensatory head and eye nystagmus in the running monkey. J. Neurophysiol. 67, 1146–1157. 1597704 10.1152/jn.1992.67.5.1146

[B49] Steinman R. (2003). Gaze control under natural conditions, in The Visual Neurosciences, Chapter 90, eds Chalupa L. M. Werner J. S. Barnstable C. J. (Cambridge, MA: MIT Press), 1339–1356.

[B50] Takei Y. Grasso R. Amorim M. A. Berthoz A. (1997). Circular trajectory formation during blind locomotion: a test for path integration and motor memory. Exp. Brain Res. 115, 361–368. 9224864 10.1007/pl00005705

[B51] Vallar G. Lobel E. Galati G. Berthoz A. Pizzamiglio L. Le Bihan D. (1999). A fronto-parietal system for computing the egocentric spatial frame of reference in humans. Exp. Brain Res. 124, 281–286. 9989433 10.1007/s002210050624

[B52] Van der Steen J. Bruno P. (1995). Unequal amplitude saccades produced by aniseikonic patterns: effects of viewing distance. Vision Res. 35, 3459–3471. 8560812 10.1016/0042-6989(95)00138-5

[B53] Vieilledent S. Kosslyn S. M. Berthoz A. Giraudo M. D. (2003). Does mental simulation of following a path improve navigation performance without vision? Cogn. Brain Res. 16, 238–249. 10.1016/S0926-6410(02)00279-3 12668233

[B54] Viviani P. Terzuolo C. (1982). Trajectory determines movement dynamics. Neuroscience 7, 431–437. 7078732 10.1016/0306-4522(82)90277-9

[B55] Wei M. Angelaki D. E. (2004). Does head rotation contribute to gaze stability during passive translations? J. Neurophysiol. 91, 1913–1918. 10.1152/jn.01044.2003 14657193

[B56] Wilkie R. M. Wann J. P. (2003). Eye-movements aid the control of locomotion. J. Vision 3, 677–684. 10.1167/3.11.3 14765952

[B57] Yoshida K. McCrea R. Berthoz A. Vidal P. P. (1982). Morphological and physiological characteristics of inhibitory burst neurons controlling horizontal rapid eye movements in the alert cat. J. Neurophysiol. 48, 761–784. 7131052 10.1152/jn.1982.48.3.761

