# Supplementary material for: Differences in gaze anticipation for locomotion with and without vision
Source: Front Hum Neurosci. 2015 Jun 8;9:312. doi: 10.3389/fnhum.2015.00312 (PMC4458691; doi:10.3389/fnhum.2015.00312)
Supplement: Supplementary file 1 [file DataSheet1.PDF]

Frontiers Human Neuroscience

Research Article
23 April 2015

Supplementary Material: Differences in gaze anticipation for locomotion with and without vision

Colas N. Authié [1,*], Pauline M. Hilt [1], Steve N'Guyen [1], Alain Berthoz [1] and Daniel Bennequin [2]

[1] *Laboratoire de Physiologie de la Perception et de l'Action, UMR 7152, Collège de France, CNRS, Paris, France*
[2] *Équipe Géométrie et Dynamique, Institut de Mathématiques de Jussieu, UMR 7586, Paris, France*

Correspondence*:
Colas Authié
Present address: Institut de la vision, UMR 7210, Université Pierre et Marie Curie-UPMC, CNRS, INSERM, 17 rue Moreau, 75012, Paris, France, colas.authie@gmail.com

1 SUPPLEMENTARY METHODS

1.1 CHOICE OF PELVIS DIRECTION AS REFERENCE FOR ANTICIPATION MEASURES

In order to study the spatial and temporal anticipation of the body segments, we choose the direction of the pelvis as the reference in the horizontal plane, and not the tangent to the trajectory of the pelvis because the direction of the pelvis is more stable than the tangent to its trajectory. The tangent oscillates around the pelvis direction (see Figure 1.A-B).

The *variability* of an angle θ varying in a time interval dt was defined by the mean square of the derivative:

$$S^{-1} = \frac{1}{T}\sqrt{\int_0^T \left(\frac{d\theta}{dt}\right)^2 dt} \tag{1}$$

The *stability* S is defined as the inverse of the variability.

We computed stability in the horizontal plane for the head direction (HD), the tangent to head trajectory (THT), the pelvis direction (PD), the tangent to the pelvis trajectory (TPT), and the dependency of these stabilities upon experimental conditions (Figure 1). A four-way ANOVA (visual condition (2) $\times$ trajectory (2) $\times$ trials (4) $\times$ stability (4)) indicated that the stability was significantly better for HD, followed by PD, then by TPT and by THT ($F(3, 27) = 99.69$, $p < 0.001$, $\eta^2 p = 0.92$, see Figure 1.C). This stability remained unaffected by the visual condition ($F(1, 9) = 0.83$, $p = 0.88$) or the trial ($F(3, 27) = 1.30$, $p = 0.29$). All considered stabilities were greater in the limaçon than in the eight shape ($F(1, 9) = 35.36$, $p < 0.001$, $\eta^2 p = 0.79$).

To summarize, independently of the visual condition, this analysis shows that the physical directions (HD, PD) are more stable than the trajectories tangents (THT, TPT). In particular, comparing gaze or head angles to TPT would introduce artificial oscillations that do not occur when referring to PD.

2 SUPPLEMENTARY RESULTS

2.1 SPATIAL ANTICIPATION OF GAZE, HEAD, SHOULDERS AND TORSO COMPARED TO THE TRAJECTORY

In order to analyze spatial anticipation, we computed the mean angles of the segments for each trial with respect to the horizontal angle of the pelvis. The results presented here also take in account shoulders and torso orientations. A four-way ANOVA revealed an effect of the visual condition ($F(1, 9) = 31.94$, $p < 0.01$, $\eta^2 p = 0.78$). The anticipation of the different segments is larger in light ($11.67 \pm 3.59°$) than in darkness ($6.07 \pm 2.44°$). An effect of the considered segment is also observed ($F(3, 27) = 89.07$, $p < 0.01$, $\eta^2 p = 0.91$). Gaze is the most anticipated segment on the trajectory ($20.1 \pm 5.5°$), followed by the head ($11.23 \pm 4.17°$), then by the shoulders ($2.94 \pm 1.94°$) and finally by the torso ($1.20 \pm 1.21°$, Figure 2.A).

A posteriori comparisons indicate a significant difference between gaze and the other three segments. Similarly, we observe a significant anticipation of the head segments on shoulders and torso. The difference between shoulders and torso is not significant.

The interaction between the two variables – visual condition, segment – is significant ($F(3, 27) = 36.89$, $p < 0.01$, $\eta^2 p = 0.80$). The anticipation of gaze and head on the pelvis is smaller in darkness (gaze: $12.67 \pm 6.05°$; head: $7.67 \pm 3.79°$) than in light (gaze: $27.53 \pm 6.91°$; head: $14.79 \pm 6.02°$). No significant difference is observed across visual conditions for the relative angles of the shoulders (light: $3.07 \pm 1.99°$; darkness: $2.83 \pm 1.91°$) and of the torso (light: $1.29 \pm 1.25°$; darkness: $1.11 \pm 1.19°$). *Post-hoc* comparisons indicate a significant difference between gaze and the head segment across visual conditions, but no effect of the visual condition is observed on anticipation of shoulders and torso. These results – less anticipation in darkness, anticipation of gaze over head, itself anticipating the other body segments – are observable for all the participants (Figure 2.C).

2.2 INDEPENDENCY OF ANTICIPATION VARIABLES

The spatiotemporal characteristics of gaze and pelvis coordination (*e.g.* the magnitude of anticipation in both spatial and temporal domains) could *a priori* vary according to both the walking speed and the curvature of the trajectories, as both factors could differ between light and darkness.

Curvature of the trajectory The mean radius of curvature (*i.e.* inverse of the curvature) of the pelvis trajectory has been calculated for each trial of each participant. A three-ways repeated measures ANOVA (visual condition (2) $\times$ trajectory (2) $\times$ trials (4)) indicated no effect of the visual condition ($F(1, 9) = 0.336$, $p = 0.58$), the trajectory radius of curvature being similar in darkness (1.24 ± 0.06 m) and in light (1.20 ± 0.05 m). The radius of curvature was not different between trials ($F(3, 27) = 2.22$, $p = 0.11$) but was without surprise larger in the limaçon (1.41 ± 0.08 m) than in the eight shape (1.03 ± 0.04 m; $F(1, 9) = 14.256$, $p < 0.005$, $\eta^2 p = 0.61$).

If the curvature does not differ across visual conditions, it does not either constitute a covariate of anticipation if averaged over a trial. However, this is not in conflict with previous observations showing an increased spatial anticipation with trajectory curvature (**Bernardin et al.**, 2012) during a trial.

Walking speed Contrarily to the curvature, the average walking speed significantly decreased in darkness (0.67 ± 0.10m/s) with respect to light condition (0.80 ± 0.14 m/s, see Results section). This factor could partlty explain the anticipation difference between visual conditions. We tested the effect of walking speed on head and gaze anticipations from correlation analyses: we found out that the correlation between spatial anticipation and speed was not straightforward (gaze anticipation independant from speed: $p > 0.25$; but head anticipation significantly correlated to speed in the dark only: $p < 0.05$). It is even more so difficult to state without any doubt that the speed could be a significant covariate of spatial anticipation depending on the visual condition.

2.3 MODELING THE ANTICIPATING BEHAVIORS IN THE DARK

To try to further characterize the difference of eye and head anticipations across the two lighting conditions, we conjectured that the anticipation in the dark was a residual of the well-known anticipation in light. It thus could be the result of two different mechanisms: the residual gaze anticipation in darkness would either correspond to the subtraction of an angular shift present in light, both for the eye and for the head; or it would match a weakening of anticipation and should be better modeled by a division of the anticipatory angles by a certain coefficient.

To challenge the two pure propositions (as it may as well be a combination of the two), we computed the expected additive residue between light and darkness on the one hand, and the expected denominator of light over darkness on the other hand. For each subject, we calculated the angular difference between anticipations in light and darkness, and averaged it over all trials. We computed the mean residue, that is the angular differences averaged over all subjects, and added it to the anticipation in darkness (giving a corrected anticipation). The challenging figure was defined as the mean deviation of the corrected anticipations from the anticipations in light, where mean deviation means averaged over all the subjects. The figure of merit for the multiplicative model is similarly calculated as the mean deviation of the individual angular ratios of light over dark, corrected by a mean coefficient, from the anticipation angles in light.

For the eye anticipation, the performance of the two models is almost identical (additive: 0.65; multiplicative: 0.69), while for the head the score of the multiplicative model is a little better (1.19) than for the subtractive model (1.68). However, the two models seem overall comparable. Thus we concluded that both models can explain the data, and were not able to discriminate between these two models.

3 SUPPLEMENTARY TABLES AND FIGURES

REFERENCES

Bernardin, D., Kadone, H., Bennequin, D., Sugar, T., Zaoui, M., and Berthoz, A. (2012), Gaze anticipation during human locomotion., *Experimental Brain Research*, 223, 1, 65–78, doi:10.1007/s00221-012-3241-2

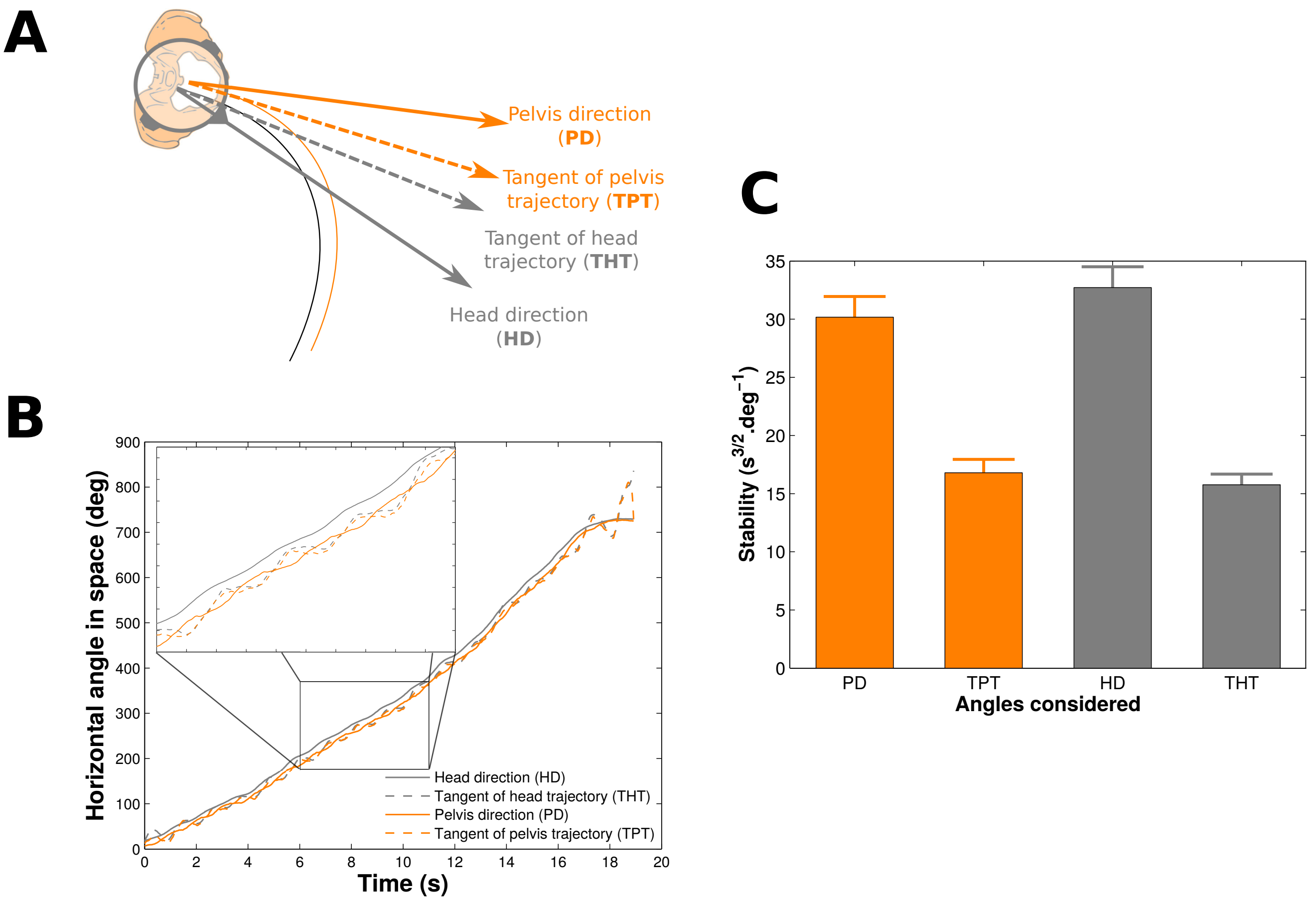

Figure 1. Reference for spatial and temporal anticipation. A. We considered angles in the horizontal plane for the head direction (HD), the tangent to head trajectory (THT), the pelvis direction (PD) and the tangent to pelvis trajectory (TPT). **B.** Horizontal angles in space for a single trial. THT and TPT are less stable than HD and PD. **C.** Average stability in the experiment. The vertical bars represent the between-participant standard deviation.

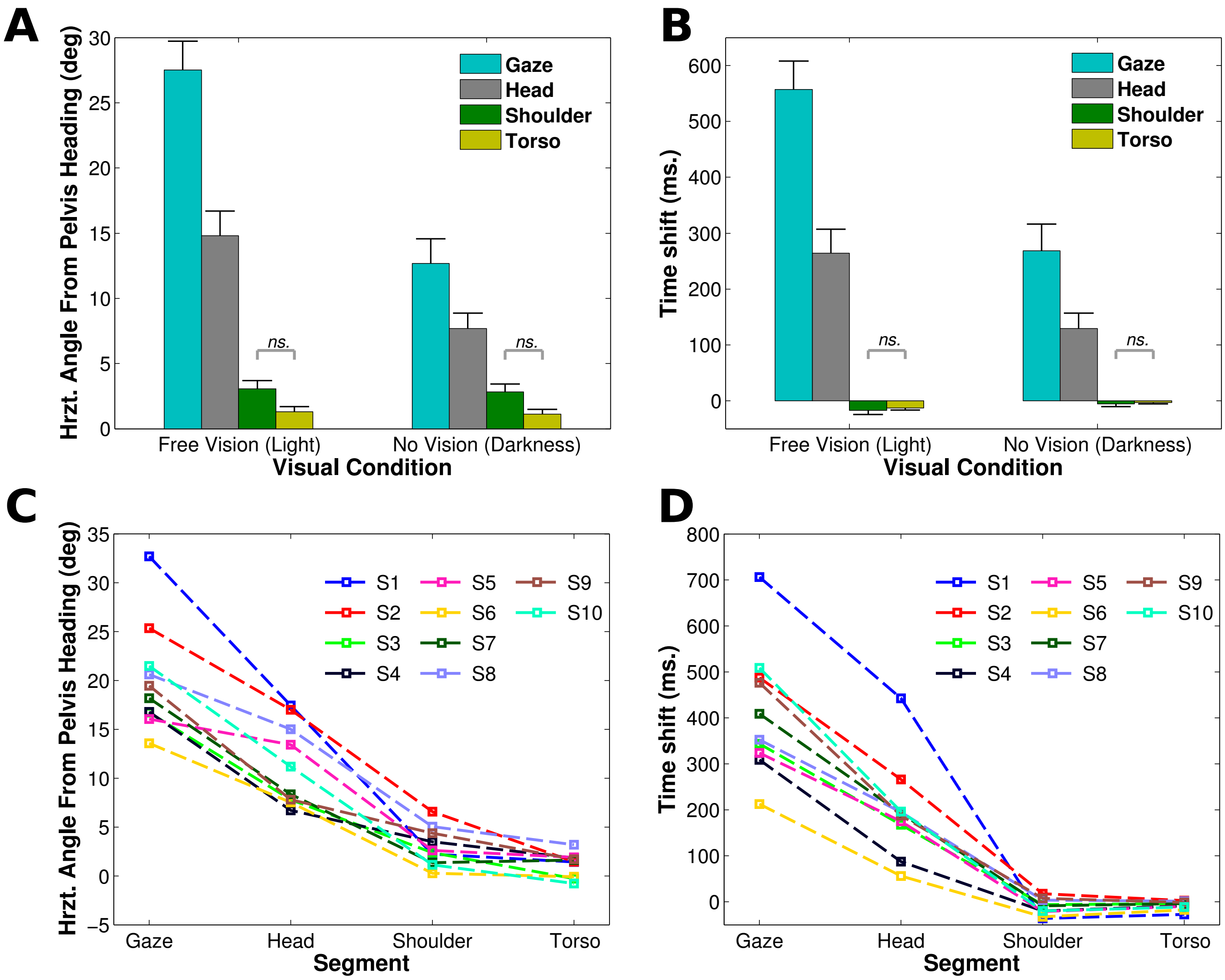

Figure 2. Spatial and temporal anticipation of the body segments. A–B. Averaged anticipations in both visual conditions are represented (left: in light, right: in darkness). Non significant differences across conditions are denoted $n.s.$ (significance threshold: $p < 0.05$). The vertical bars represent the between-participant standard deviation. **A.** Spatial anticipation of the body segments: gaze (blue), head (gray), shoulders (green) and torso (yellow) with respect to the tangent to the pelvis trajectory. **B.** Time shift of the different body segments with respect to the pelvis movement. **C–D.** Average spatial anticipation (**C**) and time shift (**D**) of the body segments for each participant. Each color corresponds to one participant.
